# Vision Sensor for Automatic Recognition of Human Activities via Hybrid Features and Multi-Class Support Vector Machine

**DOI:** 10.3390/s25010200

**Published:** 2025-01-01

**Authors:** Saleha Kamal, Haifa F. Alhasson, Mohammed Alnusayri, Mohammed Alatiyyah, Hanan Aljuaid, Ahmad Jalal, Hui Liu

**Affiliations:** 1 Department of Computer Science, Air University, Islamabad 44000, Pakistan; 232701@students.au.edu.pk; 2 Department of Information Technology, College of Computer, Qassim University, Buraydah 52571, Saudi Arabia; hhson@qu.edu.sa; 3 Department of Computer Science, College of Computer and Information Sciences, Jouf University, Sakaka 72388, Saudi Arabia; maalnusayri@ju.edu.sa; 4 Department of Computer Science, College of Computer Engineering and Sciences, Prince Sattam Bin Abdulaziz University, Al-Kharj 16278, Saudi Arabia; m.alatiyyah@psau.edu.sa; 5 Department of Computer Sciences, College of Computer and Information Sciences, Princess Nourah Bint Abdulrahman University, P.O. Box 84428, Riyadh 11671, Saudi Arabia; haaljuad@pnu.edu.sa; 6 Department of Computer Science and Engineering, College of Informatics, Korea University, Seoul 02841, Republic of Korea; 7 Cognitive Systems Lab, University of Bremen, 28359 Bremen, Germany

**Keywords:** human activity recognition, object detectors, spatio-temporal features, key body points, body pose, machine learning, human motion analysis

## Abstract

Over recent years, automated Human Activity Recognition (HAR) has been an area of concern for many researchers due to its widespread application in surveillance systems, healthcare environments, and many more. This has led researchers to develop coherent and robust systems that efficiently perform HAR. Although there have been many efficient systems developed to date, still, there are many issues to be addressed. There are several elements that contribute to the complexity of the task, making it more challenging to detect human activities, i.e., (i) poor lightning conditions; (ii) different viewing angles; (iii) intricate clothing styles; (iv) diverse activities with similar gestures; and (v) limited availability of large datasets. However, through effective feature extraction, we can develop resilient systems for higher accuracies. During feature extraction, we aim to extract unique key body points and full-body features that exhibit distinct attributes for each activity. Our proposed system introduces an innovative approach for the identification of human activity in outdoor and indoor settings by extracting effective spatio-temporal features, along with a Multi-Class Support Vector Machine, which enhances the model’s performance to accurately identify the activity classes. The experimental findings show that our model outperforms others in terms of classification, accuracy, and generalization, indicating its efficient analysis on benchmark datasets. Various performance metrics, including mean recognition accuracy, precision, F1 score, and recall assess the effectiveness of our model. The assessment findings show a remarkable recognition rate of around 88.61%, 87.33, 86.5%, and 81.25% on the BIT-Interaction dataset, UT-Interaction dataset, NTU RGB + D 120 dataset, and PKUMMD dataset, respectively.

## 1. Introduction

In computer vision, Human Activity Recognition (HAR) aims to understand the activity between individuals. Researchers have applied different machine learning algorithms to identify and understand human behaviors such as hugs and handshakes. Researchers also aim to enhance the recognition accuracy of human activity in real time, which can be beneficial for many applications, such as surveillance and security [1] and healthcare monitoring [2].

Generally, HAR systems follow one of two approaches. The first is wearable-based systems, where individuals wear devices and sensors that capture their physical movements and activities. While this approach allows for real-time monitoring by collecting a wide variety of data, which helps to provide more personalized feedback, the lack of contextual environment information might decrease the accuracy of interpreting human behaviors, especially with complex or dynamic environments. The second is vision sensor-based systems, where we utilize cameras and computer vision techniques to capture, analyze, and interpret human behaviors. By capturing detailed information about the environment, this approach provides a better understanding of the activities. However, many challenges are raised with this approach, such as lighting conditions, occlusions, and feature extraction and classification.

In computer vision and machine learning, spatio-temporal feature extraction plays an important role for HAR, as it allows for the capture of spatial and temporal information. This integration enhances the accuracy of identifying activities and differentiating between complex actions. There are many techniques for spatio-temporal feature extraction, such as 3D Pose Estimation [3], 3D Convolutional Neural Networks [4], and Graph-Based Approaches [5]. Other models, such as Support Vector Machines, also play an important role in activity classifications in HAR, as they operate on extracted spatial and temporal features from data.

In this paper, we propose an innovative approach for the automatic recognition of human activities by effectively extracting robust spatio-temporal features and utilizing multi-class Support Vector Machines (SVMs) for the classification of complex activities. Our system uniquely combines key body points and full-body features to achieve a more comprehensive and discriminative representation of activities, making it more efficient in identifying subtle differences between various human activities. The hybrid feature extraction approach allows for the integration of spatial and temporal information, capturing movement dynamics and nuances within activities. By leveraging a multi-class SVM classifier, the proposed method can manage the complexities of multiple activity classes, leading to more precise recognition even when faced with similar gestures or dynamic environmental conditions. This novel combination of hybrid feature extraction and multi-class classification serves as a pivotal advancement over existing HAR techniques, offering a balanced trade-off between feature richness and computational efficiency.

HAR systems currently struggle with diverse real-world applications. They have difficulty recognizing all the different settings, like indoor and outdoor environments, in which human activities can occur. This is compounded by the systems needing to be robust enough to handle real-world problems, like poor lighting, different viewing angles, and occlusions. These factors often lead to decreased accuracy and reliability of recognition systems, which affects the HAR system performance. However, there is an opportunity to enhance performance by utilizing a hybrid system made up of several features and a multi-class classification based on SVMs. This can give a morphologically better representation of features and a substantial likelihood of better classification in the various rendered environments of HAR systems.

To further clarify the application schemes within the vision sensor-based approach for Human Activity Recognition (HAR), it is crucial to distinguish between various targeted scenarios. For instance, in surveillance and security, HAR systems are utilized to detect suspicious behaviors such as loitering, aggression, or unauthorized access in diverse lighting conditions and crowded environments. These capabilities are critical for enhancing public safety and monitoring sensitive areas like airports, schools, and banks. In healthcare, HAR systems can monitor patients for fall detection, abnormal movements, or rehabilitation progress, requiring accurate recognition even with occlusions caused by medical equipment. Such systems play a vital role in elderly care, remote patient monitoring, and early intervention for physical or cognitive impairments.

Additionally, in smart environments and human–computer interaction (HCI), HAR systems recognize context-specific gestures for controlling devices, such as smart home appliances or industrial robotics, thereby ensuring occupant safety and improving user experience. For example, gestures can be used to turn lights on or off, adjust thermostats, or interact with virtual assistants seamlessly. In sports analytics, HAR systems can be deployed to analyze athletes’ movements, improve techniques, and prevent injuries by identifying incorrect posture or patterns. These systems also enhance training by providing real-time feedback during practice sessions.

In entertainment and gaming, HAR enhances immersive experiences in virtual reality (VR) and augmented reality (AR) by recognizing and responding to user actions. It allows for natural interaction with virtual environments, creating more engaging and personalized gaming or storytelling experiences. In education and training, HAR systems can monitor students’ gestures and activities in virtual classrooms or training simulations, ensuring effective learning engagement. For example, in skill-based training like surgery or industrial processes, HAR systems can provide feedback on accuracy and technique. By addressing these diverse applications, our proposed approach—integrating hybrid spatio-temporal feature extraction with multi-class SVM classification—is better positioned to handle the nuances and challenges of each use case, offering a highly adaptable and precise recognition system.

The contributions of this research to the field are multifaceted. Firstly, we develop a resilient HAR system capable of efficiently handling various challenging conditions encountered in both indoor and outdoor environments, such as poor lighting, different viewing angles, and intricate clothing styles. Secondly, the proposed model undergoes extensive evaluation across several benchmark datasets, including BIT, UT, NTU RGB + D 120 and PKU MMD, demonstrating its robustness and versatility in different scenarios. The evaluation results show high performance across multiple metrics—achieving high accuracy, precision, F1 score, and recall—consistently outperforming existing state-of-the-art systems in HAR. The broader impact of this research is reflected in its potential applications for real-world systems, including intelligent surveillance for security purposes, healthcare monitoring for patient activities, and intelligent environment monitoring. Additionally, the proposed approach enhances current methodologies and provides a framework that could inspire future advancements in HAR systems, particularly for applications requiring reliable and real-time recognition of complex human activities.

## 2. Related Works

Understanding and analyzing human activities plays a key role in developing efficient real-time HAR applications. In recent years, HAR has witnessed significant advancements driven by various methodologies and approaches. Researchers have been actively contributing to the development of robust HAR systems. To better understand the HAR systems, we can categorize them as wearable devices and sensor-based technology.

### 2.1. Wearable Device-Based HAR Systems

Wearable devices for HAR are electronic devices worn or attached to the body to capture the information regarding a person’s physiological and behavioral metrics. These collected data help identify various human activities and gestures. The devices include smart watches, fitness trackers, smart clothing, and wearable sensors. They include accelerometers, gyroscopes, heart rate monitors, light-emitting diodes, infrared markers, and sometimes GPS. They monitor and track movements, which allows for recognizing activities. For example, a human behavioral pattern recognition (BRP) method was proposed by Quaid et al. [6] to analyze behavioral changes in humans using multiple features. Moreover, a wearable device-based motion tracking system was proposed by Khan et al. [7] to analyze the movements of various body parts, for accurate detection of the movements that could later benefit in therapeutic decisions. Furthermore, Gochoo et al. [8] proposed a model where data are obtained utilizing hierarchical features and a multiple layer kernel sliding perceptron for adaptive learning. Similarly, S. Ashry et al. [9] developed an activity recognition system using inertial data by simply combining multiple axes from inertial measurement units (IMU) and employing that data to train hidden Markov models. In their approach, they solely rely on inertial data for recognizing motion patterns. Similarly, Chen et al. [10], in their proposed approach, attached an IR camera and an infrared emitter to a passive hand skateboard to train the device for conventional upper limb training. The device successfully trained eight people with abnormal upper limb function; after four weeks of training, the patients could move the hand skateboard along a designated figure-eight path. Also, in [11], Esfahani et al. deployed a trunk motion system (TMS) by using a printed body-worn sensor (BWS). To track the trunk movements, 12 BWS were printed and attached to the stretchable clothing.

### 2.2. Vision Sensor-Based HAR Systems

Vision sensor technology allows us to detect and measure physical properties and changes in the environment. These sensors can convert temperature, motion, pressure, light, or sound into electrical signals that can be further studied and interpreted. Vision sensor technology plays a crucial role in human activity recognition systems because of its various applications in healthcare, environmental monitoring, etc. By providing precise and real-time data, sensor technology helps in the development of intelligent systems that can monitor, analyze, and respond to dynamic conditions and activities. This enhances the system’s efficiency, safety, and overall functionality.

In sensor-based HAR systems, Ryoo and Aggarwal [12] developed a spatio-temporal relationship match, effectively localizing complex non-periodic activities by capturing local features and extracting relationships. Furthermore, Kung and Fu [13] introduced a hierarchical model where they associated hidden variables with spatio-temporal patches; it contributed to the recognition of the interacting individuals’ actions. Recent research efforts made by Jalal et al. [14] have played a vital role in the advancement of feature extraction techniques by integrating multiple feature types and proposing hybrid approaches to leverage comprehensive feature sets and classification.

Advancements in context of representation and relationship modeling have significantly improved feature classification in sensor-based HAR. For instance, context representation techniques proposed by Gaur et al. [15] focused on capturing contextual information such as environmental conditions, spatial context, temporal context, etc., surrounding human activities. Zhang et al. [16] exemplified relationship modeling techniques by focusing on relationships between interacting entities within the scene. This involves analyzing spatial and temporal relationships between individuals, groups, or objects involved in the activity. Modeling these features allows HAR to infer the underlying structure of activities and identify patterns that may not be apparent from individual features alone.

Furthermore, approaches proposed by Wang et al. [17] focus on incorporating semantic information into feature representation. This categorizes human actions in terms of semantic information such as walking, talking, or shaking hands. Overall advancements in context representation and relationship modeling have revolutionized feature classification in HAR models. By doing so, more rich and meaningful representation of human activities is obtained, leading us to more robust, precise, and reliable models.

## 3. Materials and Methods

Our proposed architecture is centered on extracting robust spatio-temporal features that effectively define each activity class. The process begins with the conversion of video frames into images, followed by a series of preprocessing techniques aimed at cleaning raw image data and isolating relevant information. This preprocessing step is designed to reduce computational costs while maintaining the integrity and quality of the data for further analysis.

The preprocessing pipeline comprises three key stages: image normalization, noise removal, and Region of Interest (ROI) extraction [18,19,20]. Image normalization scales pixel values to a standard range, enhancing the uniformity of input data and improving the model’s ability to generalize across varying conditions. Noise removal is performed using the Non-Local Means (NLM) algorithm, effectively preserving image details while eliminating extraneous noise. ROIs are then extracted by identifying human silhouettes from the background using connected component analysis. This ensures that only the relevant portions of the image are retained for subsequent processing. To further refine the isolation of subjects, the GrabCut algorithm is employed for body segmentation, providing precise separation of the silhouettes from complex or cluttered backgrounds [21,22,23,24].

Subsequent steps involve generating skeletal representations of the segmented silhouettes and extracting key points corresponding to major body parts such as the head, torso, arms, and legs. These key points enable the capture of detailed spatial and temporal features that are essential for accurately distinguishing between activity classes. The extracted features are then optimized by reducing their dimensionality in order to lower computational costs and enhance efficiency, ensuring that only the most informative and discriminative features are retained.

**Figure 1:**
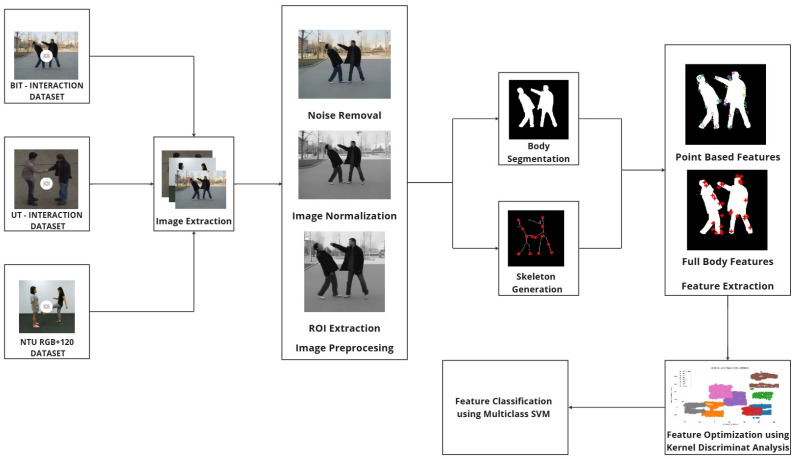
Conceptual view of proposed human activity recognition architecture.

To ensure the robustness and reproducibility of our approach, we employed a k-fold cross-validation method (with k = 5) for dataset partitioning. This method divides the dataset into five equal parts, with each subset used as a validation set once, while the remaining subsets are used for training. This iterative process not only minimizes overfitting but also ensures that every observation in the dataset is utilized for both training and validation. By adopting this strategy, we maximize the utility of the datasets and provide a more reliable estimate of the model’s performance across diverse data subsets. Figure 1 illustrates the overall architecture of our proposed model, including all preprocessing, feature extraction, optimization, and classification steps.

### 3.1. Preprocessing Stage

We applied a rigorous preprocessing pipeline to the BIT Interaction, UT Interaction, NTU RGB + D 120, and PKU MMD datasets to ensure consistency and optimize feature extraction. Video sequences were converted into frames, and keyframes were selected using histogram-based intensity analysis to reduce redundancy and computational complexity.

For noise reduction, the Non-Local Means (NLM) denoising algorithm [25] was employed, preserving important image details while eliminating extraneous noise. Image normalization followed, scaling pixel values to a standard range for consistent input to machine learning algorithms [26]. Regions of Interest (ROIs) were extracted by isolating human silhouettes using connected component analysis [27], ensuring that only relevant features were retained for further analysis. These preprocessing steps ensured that data from diverse sources and environments were uniform and optimized for input into the hybrid feature extraction and classification framework.

Initially, videos are converted to image frames, and the number of frames for each video varies. For instance, if there are 30 frames per second, it increases the system complexity, in order to reduce the complexity of our proposed architecture. We computed 15 keyframes by analyzing the histogram distribution of pixel intensities across all frames [28]. It allows us to extract keyframes with the highest intensity changes. This can be given by the following equation:

(1) $$H\left(I\right)=arg{max}_{i\in \left(1,\ldots ,N\right)}\left(\sum_{k=1}^{B}\left|H_{i}\left(k\right)\cdot H_{i-1}\left(k\right)\right|\right)$$

where $H_{i}\left(k\right)$ represents the *k*-th bin in the histogram for the *i*-th time frame. *N* is the total number of frames, and *B* is the total number of bins in the histogram. By selecting frames with the highest values of the sum of histogram bins, the keyframes with significant changes can be extracted. Once the frames are extracted from the activity videos, the next step is to remove the noise from the image. Noise removal is applied to reduce excessive noise from the images by using nonlocal means (NLM) denoising, as it preserves the image details while removing any access noise [29]. It averages the intensity of all the similar patches while preserving the important image details. It is a more robust technique, as it works well in case of high noise levels by leveraging the redundancy in image data. NLM can be mathematically explained as follows:

(2) $$NL\left[v\right]\left(i\right)=\frac{1}{C\left(i\right)}\;\sum_{j\epsilon \Omega }v\left(j\right)\cdot \;exp\left(\frac{\left\|v\left(N_{i}\right)-v\left(N_{j}\right)\right\|\begin{array}{c} 2\\ 2 \end{array}}{h^{2}}\right)$$

where *NL[v](i)* is the denoised value of the pixel *I*, *v*(*j*) is the pixel value of the pixel *j*, *N_i_* and *N_j_* are the patches or neighboring pixels centered at pixels *i* and *j*, respectively. $\left\|v\left(N_{i}\right)-v\left(N_{j}\right)\right\|\begin{array}{c} 2\\ 2 \end{array}$ is the squared Euclidean distance between the patches *N_i_* and *N_j_*, *h* is the filtering parameter that controls the degree of filtering, and Ω is the search window—the set of all *j* pixels that are considered for averaging—and *C_i_* is the normalizing factor. Once the image is denoised, we normalize the image as it scales the pixel value to a constant range. This gives a consistent scale and better generalization to improve the performance of machine learning techniques. In other words, if normalization is not applied, the features at larger scales will dominate others and lead to inaccurate assumptions. In the proposed architecture, mean normalization is applied, as it standardizes the pixel values to a mean of 0 and a standard deviation of 1 [30]. Each image is normalized using the following:

(3) $$M=\frac{I\left(x,y\right)-mean}{max-min}$$

where *I*(*x*,*y*) is the original pixel value at the position (*x*,*y*) and *mean* is the mean pixel value of the entire image. *max* is the maximum pixel value and *min* is the minimum pixel value. Lastly, the region of interest (ROI) extraction is applied, which allows us to isolate the target area of the image for further analysis. As this region contains all of the relevant information, it enhances the focus, performance, and accuracy of the system. We have achieved ROI extraction through connected components. Initially, the connected components are computed, which helps in extracting the dimensions of the connected components to detect the human silhouettes in the image. After detecting the silhouettes, they are enclosed in bounding boxes to depict the region of interest in the image. Connected components can be given by the following formula [31,32]:

(4) $$C_{i}=\left\{\left(x_{i},y_{i},w_{i},h_{i}\right)\;|\;\sum_{p=x_{i}}^{x_{i}+w_{i}}\sum_{q=y_{i}}^{y_{i}+h_{i}}f\left(p,q\right)>0\right\}$$

where *C_i_* represents each connected component, (*x_i_*, *y_i_*) is the top left corner of the bounding box, and *w_i_* and *h_i_* are the width and height of the bounding box, respectively. The double summation $\sum_{p=x_{i}}^{x_{i}+w_{i}}\sum_{q=y_{i}}^{y_{i}+h_{i}}f\left(p,q\right)$ ensures that the bounding box encompasses all the pixels (*p, q*) where the pixel intensity function $f\left(p,q\right)$ is greater than 0, indicating the presence of components. After capturing human silhouettes using connected components, these silhouettes are enclosed in bounding boxes which can be represented mathematically as follows:

(5) $${ROI}_{i}\left(x,y\right)=I_{color}\left(x_{i}\leq x<x_{i}+w_{i}\;,\;y_{i}\leq y<y_{i}\;,\;h_{i}\right)$$

**Figure 2:**
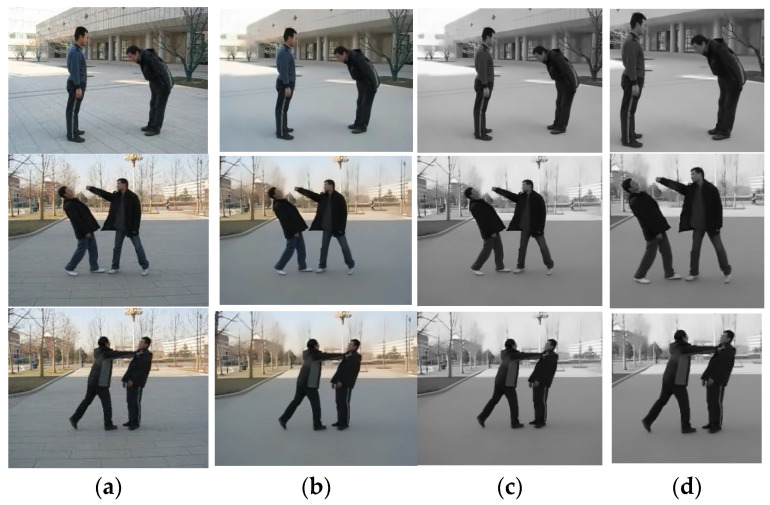
Illustration of the preprocessing stage: (**a**) original image; (**b**) noise removal; (**c**) image normalization; (**d**) ROI extraction over push activity on BIT interaction dataset.

The resulting image is the extracted ROI. Figure 2 illustrates the preprocessing stage in detail, showcasing all the steps, respectively.

### 3.2. Silhouette Segmentation

Body segmentation is a crucial step in computer vision, particularly when working on human activity recognition. In HAR, accurate segmentation of human silhouettes from images is essential for understanding social activities, gestures, and behaviors. By segmenting the silhouette, it becomes easier to analyze movements, poses, and activities between individuals. The process of silhouette segmentation typically revolves around background subtraction, region-based segmentation, or contour detection, where the main aim is to isolate the silhouettes from their surroundings; the segmented part acts as foreground and the rest is considered background. These segmented images are further used for feature extraction and, ultimately, classification, thus allowing us to develop efficient systems to interpret human behavior.

#### GrabCut Algorithm

**Figure 3:**
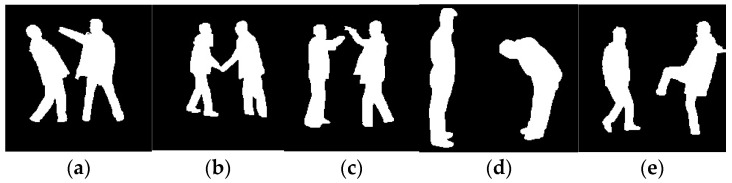
GrabCut segmentation results of (**a**) punch, (**b**) handshake, (**c**) high five, (**d**) bend, and (**e**) kick activities.

The GrabCut algorithm proposed by Rother et al. [21] is a proficient technique for foreground extraction. It offers a semi-automatic way to segment silhouettes from complex backgrounds and divides the image into two parts, i.e., foreground and background. Initially, in our proposed architecture, separate bounding boxes are initialized to detect the silhouettes in the image. Then, the GrabCut algorithm is applied to separate the foreground from the background. The GrabCut algorithm is concurrently used with a Gaussian Mixture Model (GMM) to define a rectangular area in which it labels each pixel as potential foreground and potential background. The GMM framework allows us to increase the likelihood of the observed data and allows more precise and refined silhouette segmentation. The following equation illustrates the silhouette segmentation:

(6) $$P\left(I_{i}\right|c)=\frac{1}{\sqrt{\left(2\pi \right)\sigma_{c}^{2}}}\exp\left(-\frac{{\left(I_{i}-\mu_{c}\right)}^{2}}{2\sigma_{c}^{2}}\right)$$

where $P\left(I_{i}\right|c)$ represents the probability of intensity value *I_i_* given the Gaussian component *c* (foreground or background). $\mu_{c}$ and $\sigma_{c}^{2}$ are the mean and variance of the intensity values for the Gaussian component *c*. Figure 3 illustrates the results of the GrabCut algorithm.

### 3.3. Skeleton + Key Point Generation

**Figure 4:**
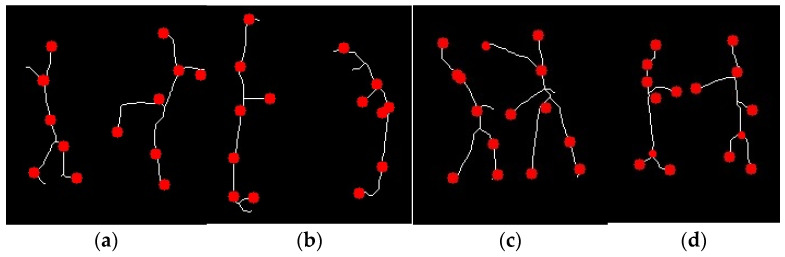
Skeleton and key point generation on (**a**) kick, (**b**) bend, (**c**) punch, and (**d**) handshake activities.

In this section, we generate a skeletal representation of our activity and define key points identifying the head, neck, torso, hands, and feet. This is done to analyze and understand the shape and structure of the silhouette in the image. To do so, we enclosed the silhouettes in three bounding boxes, i.e., an upper bounding box to identify head and neck, a middle bounding box to identify hands and torso, and lastly, a lower bounding box to identify feet. The upper bounding box helps us identify the head as the topmost point coinciding with the upper boundary of the upper bounding box and the neck as the lowest point that coincides with the lower boundary of the upper bounding box. The middle bounding box helped us identify hands by the left-most and right-most points coinciding with the left and right boundary of the middle bounding box. The torso is determined as the lowest point that coincides with the lower boundary of the middle bounding box. Similarly, feet were identified using the lower-left and -right boundaries of the bounding box. The points closest to these boundaries were identified as left and right feet, respectively [33]. Figure 4 shows a skeleton with its key points.

### 3.4. Feature Extraction

Feature extraction plays a crucial role in computer vision and machine learning algorithms, particularly when dealing with the HAR model. It captures intricate information and transforms raw data into a representative feature set. These extracted features serve as input to our machine learning algorithms for tasks such as feature optimization and classification. In this section, we will discuss the hybrid approaches integrated with our proposed architecture to extract robust spatio-temporal features for human activity recognition. The feature extraction process is divided into two subsets, where, first, point-based features are extracted based on spatial data, and then full-body features are extracted based on temporal data.

#### 3.4.1. Point-Based Features: MOvement CONtexts

**Figure 5:**
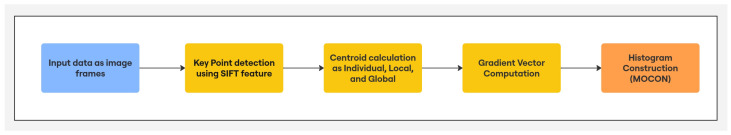
Flow chart depicting MOCON construction.

**Figure 6:**
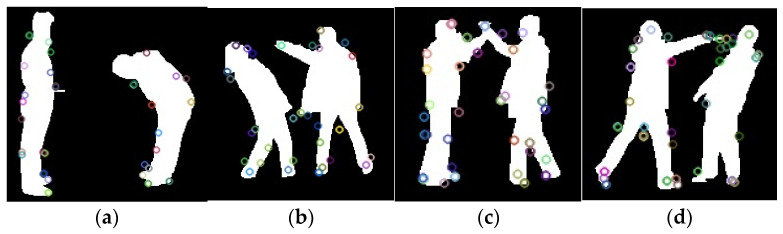
SIFT feature extraction on (**a**) bend, (**b**) punch (**c**) high five, and (**d**) push activities.

The point-based feature extraction process involves multiple stages designed to capture spatial and temporal information critical for recognizing human activities. Initially, the MOCON technique is used to represent movement contexts at the individual, local, and global levels by computing centroids and gradient vectors. These centroids summarize the spatial relationships between interacting entities in each video frame. The gradient vectors capture the motion of key points relative to these centroids, forming histograms that describe the activity’s overall motion patterns. This hierarchical approach ensures that the model captures both fine-grained and broad movement characteristics. The flow chart in Figure 5 below illustrates the flow of MOCON construction:

MOvement CONtexts (MOCONs) [34] are a spatial distribution of the local movements of the interest points. They capture the individual, local, and global movement contexts to construct a point-based feature representation of human activity. Firstly, the interest points are extracted using the SIFT algorithm along with optical flow [35]. The SIFT algorithm allows extraction of the interest points, whereas optical flow allows tracking of those interest points along the image sequence based on the smallest Euclidean distance between the SIFT features in an image sequence [36,37,38]. Figure 6 illustrates extracted SIFT features.

**Figure 7:**
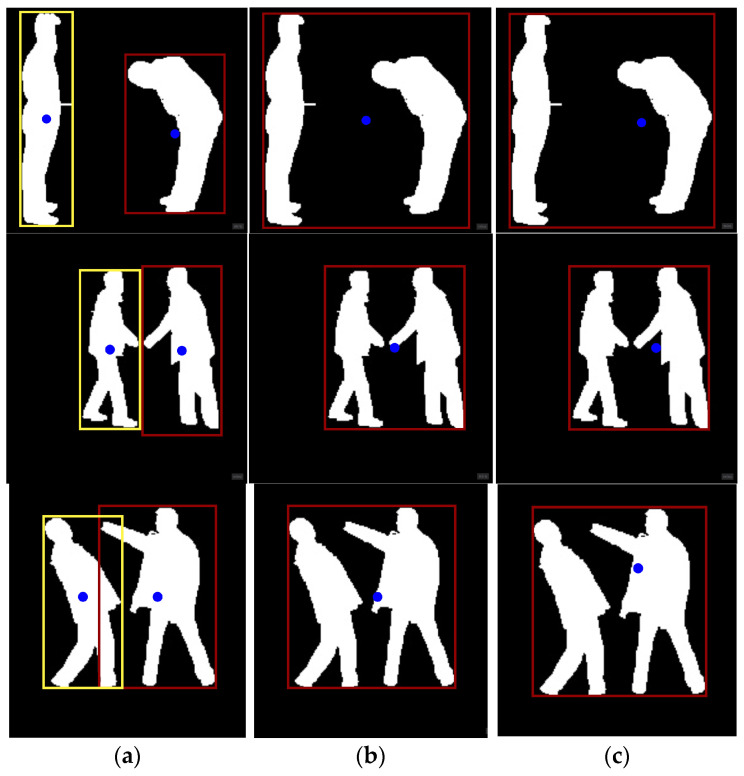
Centroid calculation: (**a**) individual; (**b**) local; (**c**) global on bend, handshake, and punch activities.

Once the interest points are extracted, the next step is to construct the MOCONs, for which the context of local movements on three different levels, i.e., individual, local, and global, are calculated [39]. Initially, three reference points *p^i^*, *p^L^* and *p^G^* are calculated. These three reference points are the centroids of the individual, local, and global movements. To obtain the individual reference point, the center position of the bounding box is calculated for each human silhouette *i* at time *t*, respectively; this can be denoted by following equation:

(7) $$P_{t}^{\left(i\right)}=\left(x_{t}^{\left(i\right)}+\sum_{k=1}^{K}w_{k}\cdot f_{k}\left(x_{t}^{\left(i\right)}\right)\;,\;y_{t}^{\left(i\right)}+\sum_{k=1}^{K}w_{k}\cdot g_{k}\left(y_{t}^{\left(i\right)}\right)\right)$$

where $x_{t}^{\left(i\right)}\;$ and $y_{t}^{\left(i\right)}$ are the initial coordinates of the silhouette *i* at time *t*, $w_{k}$ is the weight associated with the *k*-th influence factor, and $f_{k}\left(x_{t}^{\left(i\right)}\right)$ and $g_{k}\left(y_{t}^{\left(i\right)}\right)$ are functions that model the influence of the *k*-th factor on the *x*, *y* axes. Similarly, the local centroid $P_{t}^{L}$ is calculated based on the centers of both human silhouettes *i* at time *t;* mathematically, we can represent this as follows:

(8) $$P_{t}^{L}=\frac{1}{N\left(t\right)}\sum_{i=1}^{N\left(t\right)}P_{t}^{\left(i\right)}$$

where *N(t)* is the number of human silhouettes in a frame. Lastly, the global centroid *P^G^* is calculated as the average centroid for the human silhouettes over the entire image sequence. Whereas the local centroids are calculated for the centers of human silhouettes on each frame, the global centroid is calculated once for the entire sequence. We can mathematically represent the global centroid as follows:

(9) $$P^{G}=\frac{1}{T}\;\sum_{t=1}^{T}P_{t}^{L}\;$$

where *T* is the number of frames in an image sequence. Figure 7 gives a graphical representation of average centroid calculation.

The next step is to calculate gradients between the interest points and the centroids, respectively. The following equation illustrates how gradients for each local movement are calculated.

(10) $$g_{t}^{\left(I,i\right)}=\left\{P_{t}^{\left(i\right)}-q_{t}^{\left(i,j\right)}\;\left|j=1,\;2,\;\ldots ,N_{i}\right|\right\}$$

(11) $$g_{t}^{L}=\left\{P_{t}^{L}-q_{t}^{j}\;|\;j=1,\;2,\;\ldots ,N_{p}\right\}$$

(12) $$g_{t}^{G}=\left\{P^{G}-q_{t}^{j}\;|\;j=1,\;2,\;\ldots ,N_{p}\right\}$$

where $g_{t}^{\left(I,i\right)}$ represents a gradient vector between the individual centroid and the interest points, $g_{t}^{L}$ represents the gradient vector local centroid and the interest points, and $g_{t}^{G}$ represents the gradient vector between the global centroid and the interest points. $q_{t}^{\left(i,j\right)}$ is the *j*-th interest point associated with the *i*-th human silhouette at time t, $N_{i}$ is the number of interest points for the *i*-th human silhouette, and $N_{p}$ is the total number of interest points at time t. MOCONs are histogram-based representations used to capture movement context around certain reference points in a video sequence. For each image sequence, three different MOCONs are constructed, i.e., individual, local, and global. Each MOCON consists of *Hb* number of bins representing ranges of different angles. We can define each MOCON as follows:

(13) $$\lambda_{A_{s}}^{I},i=\left[h_{I,i}\left(1\right),\ldots ,h_{I,i}\left(N_{B}\right)\right]$$

(14) $$\lambda_{A_{s}}^{L}=\left[h_{L}\left(1\right),\ldots ,h_{L}\left(N_{B}\right)\right]$$

(15) $$\lambda_{A_{s}}^{G}=\left[h_{G}\left(1\right),\ldots ,h_{G}\left(N_{B}\right)\right]$$

where each element, i.e., $h_{I,i}\left(l\right)$, $h_{L}\left(l\right)$, and $h_{G}\left(l\right)$ corresponds to the *l*-th bin of the histogram. To calculate a histogram, the magnitude of each gradient vector is calculated, where each bin is populated based on the magnitude. The gradient vector captures the motion information between the reference point, i.e., individual, local, and global, and the interest points respectively. We can calculate the value of *l*-th bis as follows:

(16) $$h_{A_{S}}^{I},i\left(l\right)=\sum_{\forall j,t\epsilon A_{S}}mag\left(g_{t}^{I,i}\left(j\right)\cdot \delta \left(ang\left(g_{t}^{I,i}\left(j\right)\right)\right),\;range\left(l\right)\right)$$

(17) $$h_{A_{S}}^{L}\left(l\right)=\sum_{\forall j,t\epsilon A_{S}}mag\left(g_{t}^{L}\left(j\right)\cdot \delta \left(ang\left(g_{t}^{L}\left(j\right)\right)\right),\;range\left(l\right)\right)$$

(18) $$h_{A_{S}}^{G}\left(l\right)=\sum_{\forall j,t\epsilon A_{S}}mag\left(g_{t}^{G}\left(j\right)\cdot \delta \left(ang\left(g_{t}^{G}\left(j\right)\right)\right),\;range\left(l\right)\right)$$

where $mag\left(\cdot \right)$ returns the magnitude of the gradient vector, $ang\left(\cdot \right)$ returns the angle of the gradient vector, and $\delta \left(\left(ang\left(g\right)\right),range\left(l\right)\right)$ is an indicator function that returns 1 if the angle of the gradient vector lies within the angle range of *l*-th bin; otherwise, 0. A polar bin is used as a reference point, where each bin has the same size of angle range. The illustration below showcases the point-based features. Figure 8 is graphical representation of gradient vector of individual, local and global centers respectively.

#### 3.4.2. Full-Body Features

This section explores the hybrid feature extraction algorithm developed for analyzing full-body features in activity recognition. Although spatial features are essential for identifying activities, they often struggle with ambiguity, particularly when differentiating between visually similar activities. To mitigate this issue, our model incorporates temporal features, providing enhanced flexibility in accurately detecting and categorizing activities. Temporal features are derived by analyzing intensity changes across frames, focusing on identifying significant changes in movement or posture. The starting and ending frames of these changes are then classified into distinct categories based on the nature of the transitions observed.

Start features are defined by continuous silhouette pixel values that share similar starting frames but differ in their final frames. These features are essential in recognizing activities that initiate from the same state but diverge as the motion progresses [40]. Start features can be mathematically expressed as

(19) $$start\left(r_{1},r_{2}\right)\;↔initial\;\left(r_{1}\right)=initial\;\left(r_{2}\right)$$

In this equation, *r*_1_ and *r*_2_ represent pixels or pixel groups, and the variable *initial* denotes the starting frames of these pixel values.

Before features refer to pixel values that may or may not start at the same time but certainly end at different times, with one ending before the other. These features are crucial for detecting activities where one motion concludes before another begins [41]. The mathematical representation of before features is given by

(20) $$before\left(r_{1},r_{2}\right)\;↔final\;\left(r_{1}\right)<final\;\left(r_{2}\right)$$

Here, *r*_1_ and *r*_2_ are pixel values, with *final* representing the ending frames, where *r*_1_ concludes before *r*_2_.

Meet features occur when the pixel values of two activities coincide at a specific point in time, meaning the end of the first-pixel value coincides with the start of the second [42]. This type of feature is vital in recognizing activities where one action directly follows another. Mathematically, meet features can be described as

(21) $$meet\left(r_{1},r_{2}\right)\;↔final\;\left(r_{1}\right)=initial\;\left(r_{2}\right)$$

**Figure 9:**
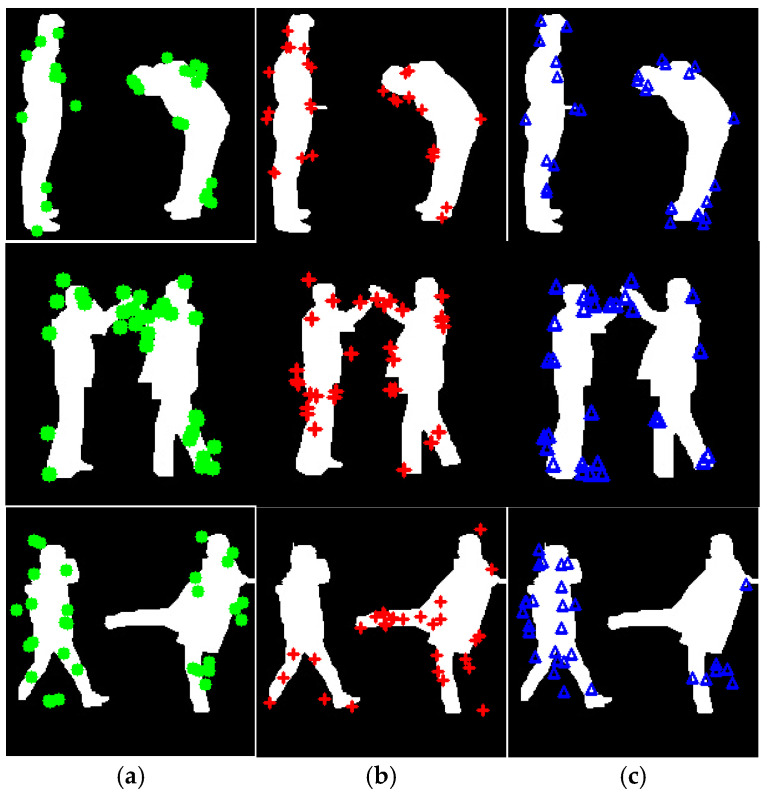
Illustration of (**a**) start, (**b**) before, (**c**) meet features on bend, high five, and kick activities.

In this equation, *r*_1_ represents the pixel value that ends precisely when the pixel value *r*_2_ begins. Figure 9 illustrates these features.

Identical features are those in which the pixel values at the initial frame and the final frame remain unchanged. These features are critical in scenarios where the activity begins and ends in a similar state, providing a consistent visual signature across the frames [43]. Mathematically, identical features can be expressed as

(22) $$identical\left(s_{1},s_{2}\right)↔start\left(s_{1}\right)=start\left(s_{2}\right)\;and\;end\left(s_{1}\right)=end\left(s_{2}\right)$$

Here, s_1_ and s_2_ represent pixels or pixel groups, and the equation highlights that the pixel values at the start and end of the frames are equivalent.

Enclosed features are characterized by the occurrence of one pixel’s timeframe entirely within the timeframe of another pixel. This category of features is important for identifying activities where a smaller or secondary motion is encapsulated within a larger, overarching motion [44]. The mathematical representation of enclosed features is given by

(23) $$enclosed\left(s_{1},s_{2}\right)↔start\left(s_{1}\right)>start\left(s_{2}\right)\;and\;end\left(s_{1}\right)<end\left(s_{2}\right)$$

In this equation, s_1_ represents a pixel with a shorter duration, completely enclosed within the timeframe of s_2_, which has a longer duration.

Double features are defined by the continuous overlap of one pixel’s data over the timeframe of another pixel, without being entirely enclosed. This type of feature is critical in detecting complex activities where multiple movements occur concurrently but are not fully contained within one another [45]. Double features can be mathematically expressed as follows:

(24) $$double\left(s_{1},s_{2}\right)↔start\left(s_{1}\right)<start\left(s_{2}\right)\;and\;end\left(s_{1}\right)>end\left(s_{2}\right)$$

Here, s_1_ and s_2_ are pixel values where s_1_ starts after s_2_ has started and ends before s_2_ has ended, indicating overlapping yet distinct features.

**Figure 10:**
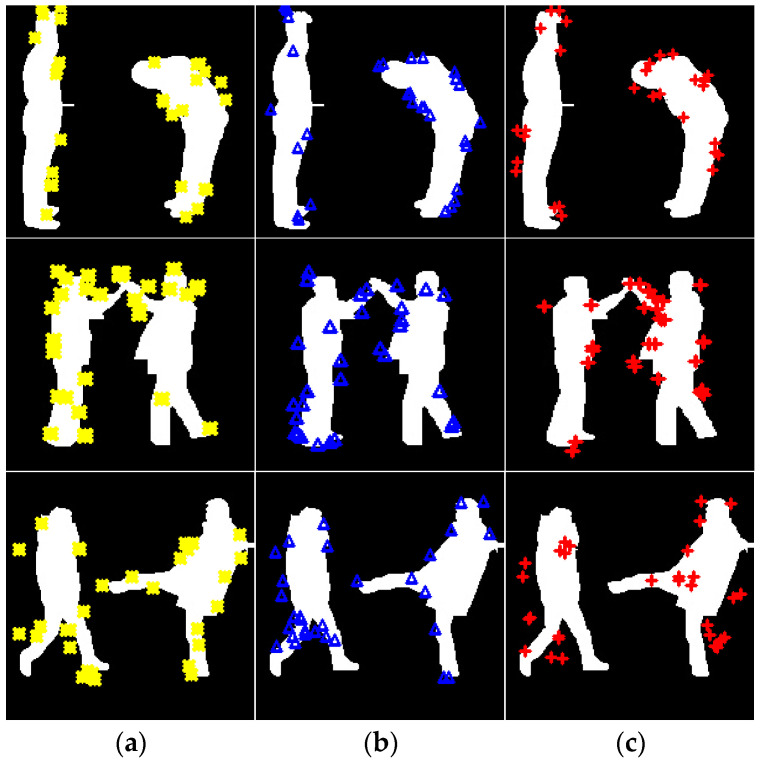
Illustration of (**a**) Identical, (**b**) enclosed, (**c**) double features on bend, high five, and kick activities.

Figure 10 illustrates the temporal features extracted from full-body silhouettes during a bend, high five, kick activity, with yellow representing identical features, blue representing enclosed features, red representing double features respectively. Figure 10 visualization aids in understanding the distribution and activity of these features across a sequence of frames.

### 3.5. Feature Optimization Using Kernal Discriminant Analysis

Feature optimization plays a very vital role in machine learning and data analysis, as it aims to improve the efficiency and effectiveness of machine learning models. By applying feature optimization, we convert high-dimensional feature data into lower-dimensional feature data. This optimized feature representation enhances the system’s robustness and cost-effectiveness, thus making the system more accurate efficient, and resilient.

Once all the intricate features are extracted, they are optimized to convert them from high-dimensional features to low-dimensional features. To accurately depict these intricate, multi-dimensional features, Kernel Discriminant Analysis (KDA) is employed. KDA converts nonlinear data from a space with a high number of dimensions to a space with fewer dimensions, facilitating class separation by efficiently recognizing detailed patterns and connections present in the data. Mathematically, this transformation can be expressed by a modified eigenvalue problem:

(25) $$\left(KHK+\gamma I\right)w=KHBKw\Lambda$$

where *K* is the kernel matrix, *w* contains the eigenvectors, Λ represents the eigenvalues, *B* is the matrix of class labels, and γI is a regularization term to ensure numerical stability. Additionally, the discriminant criterion for KDA is often defined to maximize the ratio of the between-class scatter to the within-class scatter in the kernel-induced feature space:

(26) $$max\frac{w^{T}KHBKw}{w^{T}\left(KHK+\gamma I\right)w}$$

**Figure 11:**
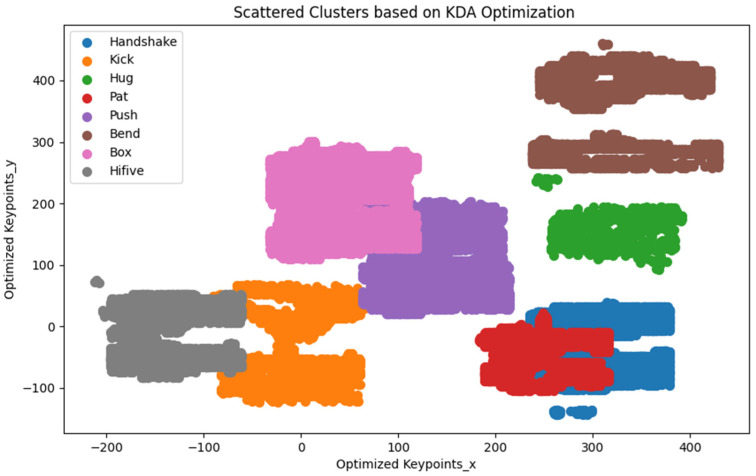
Feature optimization using kernel discriminant analysis.

This formulation allows KDA to identify the most discriminative features, thereby enhancing the model’s ability to differentiate between classes The use of KDA in this context ensures that even subtle differences between classes are captured, enabling robust and reliable classification in multi-dimensional feature spaces. The following graph illustrates the optimized classes using kernel discriminant analysis. The figure below (Figure 11) illustrates the results of applying KDA, highlighting how this approach effectively captures the complexity of the activity classes, leading to improved classification accuracy.

### 3.6. Feature Classification Using Multi-Class Support Vector Machine

Feature classification involves categorizing data based on its features to facilitate analysis and decision-making. It is done to assign labels to data points by analyzing their attributes, enabling systems to make predictions or identify patterns. This process helps in organizing and interpreting large datasets by grouping similar items. Classification is crucial in tasks like image recognition, spam detection, and medical diagnosis. It enhances the ability to make informed decisions based on the characteristics of the data. A multiclass Support Vector Machine (SVM) is an extension of the traditional binary SVM that can handle classification tasks involving more than two classes. Unlike binary SVMs, which separate data into two categories, multiclass SVMs employ strategies like one-vs.-one or one-vs.-all to partition data into multiple classes. The one-vs.-one approach constructs a classifier for every pair of classes, while the one-vs.-all method builds a classifier for each class against all others. These individual classifiers are then combined to make a final prediction. Mathematically, for a given dataset {*x_i_, y_i_*} where $x_{i}\epsilon R^{n}$ represents the feature vector and $y_{i}\epsilon \;\left\{1,2,\ldots ,K\right\}$ represents the class label, the decision function in a multiclass SVM can be represented as:

(27) $$f\left(x\right)={arg}_{j\in \left\{1,2,\ldots ,K\right\}}\max\left(w_{j}\cdot \;x+b_{j}\right)$$

**Figure 12:**
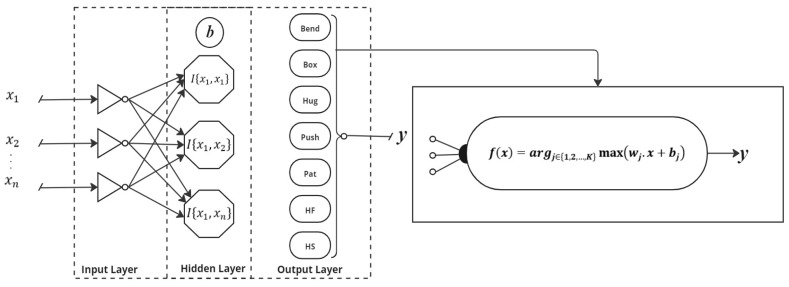
Block diagram of multiclass Support Vector Machine.

Here, *w_j_* and *b_j_* are the weight vector and bias term associated with the *j*-th class, and the classifier assigns the class label with the highest decision function value to the input *x*. Multiclass SVMs aim to find optimal hyperplanes in high-dimensional space to maximize the margin between different classes, ensuring accurate classification even in complex datasets. This approach is widely used in applications such as image and speech recognition, where data need to be categorized into more than two distinct groups. Despite its complexity, multiclass SVMs are valued for their robustness and high performance in handling multiclass classification problems. Figure 12 provides block diagram of multiclass Support Vector Machine.

## 4. Experimental Results

Our proposed model was implemented using Python 3.9 on a 64-bit Intel Core i7 PC running Windows 10 for system evaluation and training. The computer is equipped with a 5 GHz CPU and 8 GB of RAM (random access memory). In the experimental section, we illustrated the computed results and made comparisons with state of art techniques.

### 4.1. Dataset Description

#### 4.1.1. BIT—Interaction Dataset

**Figure 13:**
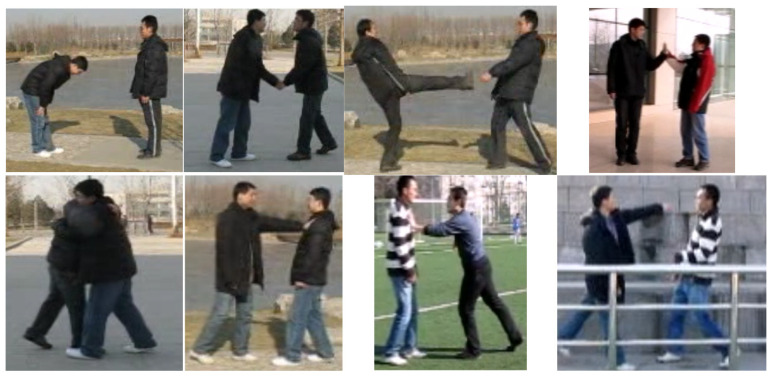
BIT—Interaction dataset with eight distinct classes.

The commonly available BIT—Interaction dataset for public use consists of eight distinct classes, i.e., handshake, hug, pat, bend, punch, push, high five, and kick. The dataset consists of dynamic indoor and outdoor settings with different backgrounds. Each activity class consists of 50 videos having a total of 400 video sequences. The video sequences involve two interacting actors; however, other pedestrians, buildings, cars, and trees are also seen as obstructions. Figure 13 illustrates some instances of the BIT—Interaction dataset.

#### 4.1.2. UT—Interaction Dataset

**Figure 14:**
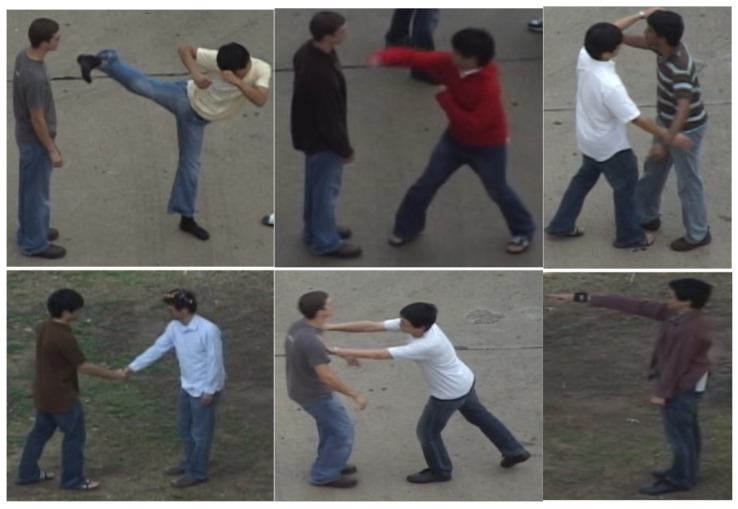
The UT—Interaction dataset with six distinct classes.

A publicly available UT—Interaction dataset consists of six interaction classes, i.e., handshake, hug, kick, push, point, and punch. The subjects in the dataset are in 15 different-colored attires with different backgrounds. The dataset consists of 20 video sequences per interaction, where each video is 720 × 480 frames per second. The video sequences are divided into subcategories: 1—in the parking lot with a static background with slight variation in zoom levels; 2—in a lawn with a dynamic background with slight movement and camera jitters. Figure 14 illustrates instances of the UT—Interaction dataset.

#### 4.1.3. NTU RGB + D 120 Interaction Dataset

**Figure 15:**
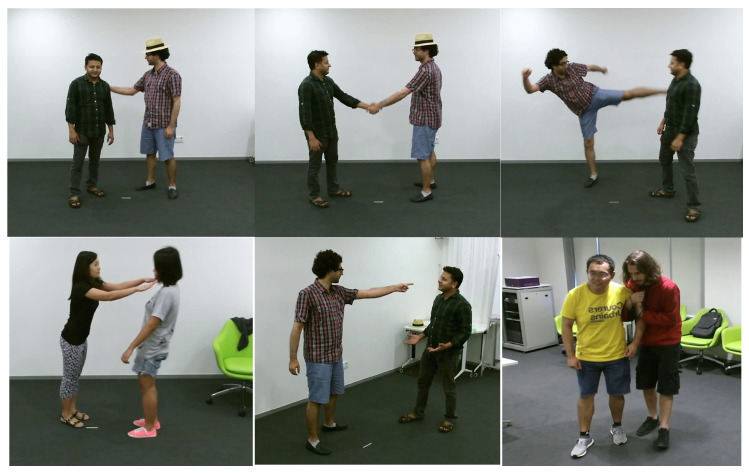
The NTU RGB + D 120 Interaction dataset with eight distinct classes involving mutual interaction.

The NTU RGB + D 120 dataset represents a substantial repository of RGB and depth-based data concerning the recognition of human activities. This dataset is composed of 114,480 video samples spanning 120 distinct action categories derived from 40 participants. These activities are segmented into three main clusters: 82 routine actions (e.g., wiping face, flipping a coin, and reading), 12 actions related to health (such as sneezing, neck discomfort, and falling), and 26 interactive actions (encompassing punching, kicking, and embracing). The acquisition of data was carried out utilizing three cameras situated at differing horizontal perspectives: −45°, 0°, and +45°. The dataset delivers a variety of information modalities for the depiction of actions, encompassing depth maps, 3D skeletal joint coordinates, RGB frames, and infrared sequences. Figure 15 illustrates instances of the NTU RGB + D 120 dataset.

#### 4.1.4. PKU MMD Interaction Dataset

**Figure 16:**
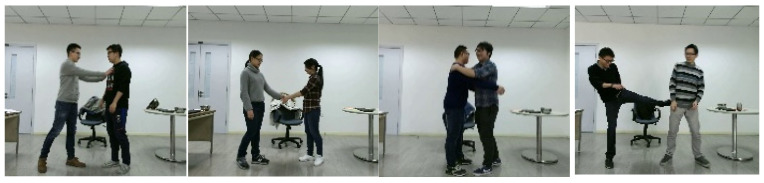

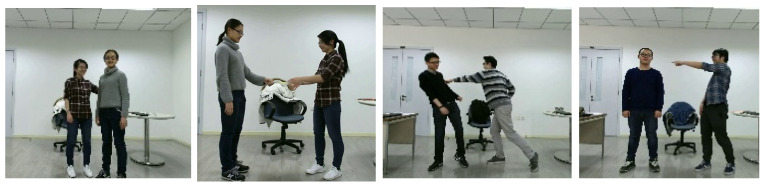
The PKU MMD Interaction dataset with eight distinct classes involving mutual interaction.

The PKU-MMD dataset is a large-scale resource for action detection and multi-modality analysis using the Kinect v2 sensor, comprising two phases. Phase 1 includes 1076 long video sequences with 51 action categories performed by 66 subjects across three camera views, resulting in nearly 20,000 action instances and 5.4 million frames at 30 FPS. These sequences feature 41 daily actions (e.g., drinking, waving) and 10 interaction actions (e.g., hugging, shaking hands). Phase 2 adds 1009 shorter sequences with 41 categories performed by 13 subjects, averaging 7 action instances per video. The dataset provides five modalities: depth maps, RGB images, skeleton joints, infrared sequences, and RGB videos, enabling comprehensive analysis. This makes it a valuable tool for continuous action detection and multimodal research. Figure 16 illustrates instances of the PKU MMD dataset.

### 4.2. Experimental Evaluation

To evaluate the performance of our proposed architecture, we conducted experiments on four benchmark datasets: BIT-Interaction, UT-Interaction, NTU RGB + D 120, and PKU-MMD. Initially, the model was tested on BIT, UT, and NTU datasets to assess its effectiveness in diverse environments and activity types. Later, we included the PKU-MMD dataset, which presents more challenging scenarios with occlusions and varying camera angles, to further test the model’s robustness. Key performance metrics such as mean recognition accuracy, precision, recall, and F1 score were computed to evaluate the system.

For feature classification, we opted to use a multi-class Support Vector Machine (SVM) due to its robustness in handling high-dimensional feature spaces and its proven effectiveness in distinguishing between complex activity classes with limited training data. While deep learning models, particularly convolutional neural networks (CNNs), are widely used in HAR, they often require large-scale datasets and substantial computational resources for optimal performance. In contrast, our approach using a multi-class SVM demonstrated a balanced trade-off between computational efficiency and classification accuracy, making it more suitable for real-time applications and environments with limited data availability.

Furthermore, we conducted additional experiments comparing our SVM-based classifier with a deep learning model (specifically a CNN-based approach). The results showed that while the deep learning model achieved comparable accuracy, it required significantly more computational resources and training time. On the other hand, our SVM classifier, combined with efficient feature extraction techniques, yielded high recognition rates (88.61% on BIT, 87.33% on UT, and 86.5% on NTU RGB + D 120) with lower computational costs. Thus, the choice of SVM was justified by its ability to provide high accuracy while maintaining efficiency, especially in scenarios with constrained hardware resources.

This experimental evaluation not only confirms the robustness of our proposed system but also highlights its practical applicability in environments where computational efficiency and scalability are critical. Table 1, Table 2, Table 3 and Table 4 show Confusion matrix on each dataset respectively.

**Figure 17:**
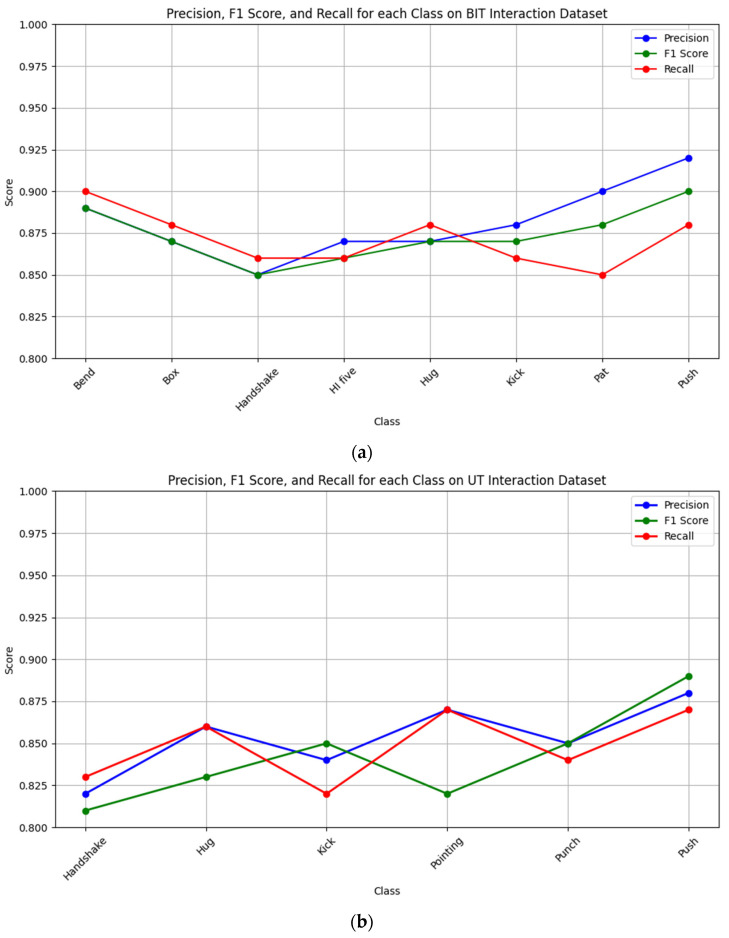

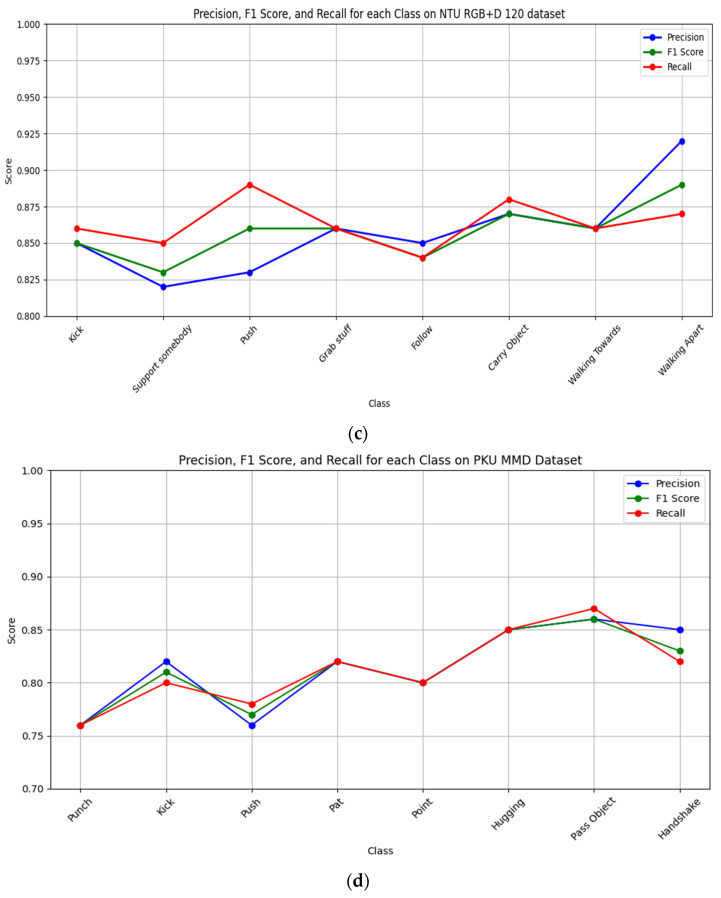
Line graph displaying F1 score, Recall, and Precision on (**a**) BIT interaction, (**b**) UT interaction, (**c**) NTU RGB + D 120, and (**d**) PKU MMD dataset.

In Figure 17, line graphs were used to visualize the F1 score, recall, and precision across the BIT Interaction, UT Interaction, NTU RGB + D 120, and PKU MMD datasets. This visualization highlights the comparative performance of the proposed model, providing a clear overview of its effectiveness across diverse datasets and performance metrics.

In analyzing the results, it was observed that certain activity classes, such as “hug” and “handshake”, demonstrated lower precision and recall compared to other activities. These misclassifications can be attributed to the inherent similarities in gestures and overlapping movement patterns, which make distinguishing between these classes challenging. Additionally, occlusions, such as partial body overlap during interactions, and variations in execution styles across different datasets, contribute to these errors. For instance, activities performed in dynamic or cluttered backgrounds further exacerbate the difficulty by introducing noise during feature extraction. Understanding these limitations highlights potential areas for improvement, such as enhancing feature discrimination through advanced techniques like spatio-temporal attention mechanisms or refining preprocessing steps to handle occlusions and noise more effectively. This analysis provides a foundation for future enhancements to the system’s robustness and accuracy.

Furthermore, we comprehensively evaluated our proposed architecture with other accepted state-of-the-art techniques. This evaluation was especially concerned with determining the average recognition accuracy in human activity recognition. The results, in Table 5, provide a thorough comparison with the state-of-the-art methods now in use. Table 6 illustrated the performance and computational comparison of multiclass SVM and deep learning across datasets. These results show a significant performance improvement, which we attribute to our proposed model.

## 5. Discussion and Practical Implications

The results demonstrate the effectiveness of the proposed hybrid feature extraction method and multi-class SVM in addressing key challenges in Human Activity Recognition (HAR). The model consistently achieved high accuracy across diverse datasets, showcasing its robustness in handling complex activity recognition tasks in varied settings.

When contextualized with prior works, such as Ryoo and Aggarwal’s spatio-temporal relationship methods or Jalal et al.’s hybrid approaches, our system advances the field by combining key body points with full-body features to achieve a more comprehensive representation of human activities. Unlike earlier methods that struggled with challenges like occlusions or dynamic backgrounds, our approach demonstrated improved resilience under these conditions.

The practical implications of these contributions are significant. In surveillance, the system’s capability to accurately identify complex interactions, even under poor lighting or occlusion, enhances security applications. In healthcare, the model’s adaptability to diverse datasets underscores its potential for monitoring patient activities, detecting falls, or analyzing rehabilitation progress. Moreover, the scalability and computational efficiency of our approach make it suitable for integration into real-time applications within resource-constrained environments.

Future work should focus on incorporating deep semantic features and leveraging advanced temporal models, such as transformers, to further enhance recognition accuracy. Additionally, the integration of multi-modal sensor data and privacy-preserving methods, such as federated learning, could broaden the applicability of HAR systems while addressing ethical concerns related to data use in sensitive domains.

## 6. Conclusions

In recent years, the domain of automated Human Activity Recognition (HAR) has garnered extensive attention due to its potential applications across diverse fields such as surveillance, healthcare, and human–computer interaction. Despite notable advancements, HAR systems continue to face limitations related to varying environmental conditions, occlusions, and activity similarities, which can degrade recognition accuracy. This study proposed a vision sensor-based HAR framework employing a hybrid feature extraction method integrated with a multi-class Support Vector Machine (SVM). Through rigorous feature extraction that captures both spatial and temporal nuances and a classifier well suited for complex activity differentiation, our system achieved recognition accuracies of approximately 88.61%, 87.33%, 86.5%, and 81.25% on the BIT-Interaction, UT-Interaction NTU RGB + D 120, and PKU MMD datasets, respectively.

The robustness of the proposed HAR model is validated through metrics such as precision, F1 score, and recall, demonstrating its resilience across diverse datasets and activity types. By combining key body points with comprehensive full-body features, the model addresses common challenges in real-world HAR applications, such as viewpoint variability and lighting inconsistencies. The proposed framework thus contributes significantly to the field by delivering a more accurate, adaptable, and computationally efficient solution for complex activity recognition. Our findings indicate that hybrid approaches incorporating multi-level feature extraction and classification can provide a reliable foundation for HAR systems in environments that demand precision and real-time responsiveness.

Building on the insights gained from this study, future research should explore incorporating additional feature types, such as deep semantic features or context-aware attributes, to enhance the model’s recognition capabilities in more complex environments. Moreover, optimizing the system for lower computational costs without compromising accuracy remains a crucial direction, especially for real-time applications on resource-constrained devices. Future work could also investigate advanced temporal models, like transformers, to better capture sequential dependencies in activities. Additionally, integrating multi-modal sensor data and privacy-preserving techniques, such as federated learning, can enhance robustness while ensuring data privacy, making the system more suitable for sensitive applications in healthcare and surveillance.

## Figures and Tables

**Figure 8:**
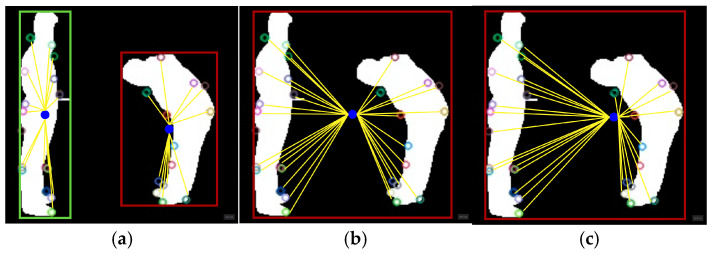
Gradient vector: (**a**) individual; (**b**) local; (**c**) global on bend activity.

**Table 1:** Confusion matrix of individual class over BIT-Interaction Dataset.

Class	Bend	Punch	Handshake	High Five	Hug	Kick	Pat	Push
Bend	90	3	2	1	2	1	1	0
Punch	2	88	3	2	1	1	2	1
Handshake	2	2	87	2	2	2	1	2
High five	1	1	2	89	2	2	2	1
Hug	2	1	2	3	88	1	2	1
Kick	1	2	3	2	2	88	1	1
Pat	1	1	2	3	2	1	89	1
Push	2	2	2	2	2	1	1	88
Mean Recognition Accuracy = 88.61%								

**Table 2:** Confusion matrix of individual class over UT-Interaction Dataset.

Class	Handshake	Hug	Kick	Pointing	Punch	Push
Handshake	87	2	2	3	3	3
Hug	3	86	3	3	2	3
Kick	2	2	89	2	2	3
Pointing	3	3	2	87	3	2
Punch	2	3	3	3	88	1
Push	3	3	2	2	3	87
Mean Recognition Accuracy = 87.33%						

**Table 3:** Confusion matrix of individual class over NTU RGB + D 120 Dataset.

Class	Kick	Support Somebody	Push	Grab Stuff	Follow	Carry Object	Walking Towards	Walking Apart
Kick	86	2	3	2	2	2	2	1
Support somebody	2	85	3	3	2	2	2	1
Push	2	2	89	2	1	1	2	1
Grab stuff	2	3	2	86	2	2	2	1
Follow	3	3	3	2	84	2	2	1
Carry Object	2	2	2	1	1	89	2	1
Walking towards	2	2	2	2	3	2	86	1
Walking Apart	2	2	2	2	2	2	1	87
Mean Recognition Accuracy = 86.50%								

**Table 4:** Confusion matrix of individual class over PKUMMD Dataset.

Class	Punch	Kick	Push	Pat	Point	Hugging	Pass Object	Handshake
Punch	76	2	4	3	5	4	2	4
Kick	3	80	5	3	3	2	2	2
Push	6	2	78	4	4	2	2	2
Pat	3	3	4	82	2	2	2	2
Point	5	3	4	4	80	2	1	1
Hugging	2	2	2	3	2	85	2	2
Pass Object	1	1	2	2	3	2	87	2
Handshake	3	2	3	2	2	4	2	82
Mean Recognition Accuracy = 81.25%								

**Table 5:** Comparison of datasets with SOTA methods.

Authors	Recognition Accuracy			
	BIT Interaction	UT Interaction	NTU RGB + D 120	PKU MMD
DMMTS [46]	85.16	-	-	-
DLM [47]	82	-	-	-
IP [48]	75	-	-	-
IBo WM [49]	-	85	-	-
STR Match Kernal [50]	-	70.8	-	-
MTSSVM [51]	-	86.6	-	-
STA—LSTM [52]	-	-	81.2	-
TS—LSTM [53]	-	-	74.60	-
Multitask Deep Learning [54]	-	-	85.5	-
STA LSTM [55]	-	-	-	44.4
JCRRNN [56]	-	-	-	53.3
Skeleton boxes [57]	-	-	-	54.8
Proposed	**88.61**	**87.34**	**86.91**	**81.25**

**Table 6:** Performance and computational comparison of multiclass SVM and deep learning across datasets.

Datasets	Recognition Accuracy		Execution Time (s)	
	Multiclass SVM	Deep Learning	Multiclass SVM	Deep Learning
BIT Interaction	88.61%	83.21%	19,064.93	29,084.56
UT interaction	87.33%	82.92%	18,521.73	25,033.55
NTU RGB D 120	86.50%	81.22%	25,095.98	35,042.23
PKU MMD	81.25%	84.16%	24,906.30	31,021.12

## Data Availability

Both datasets are publicly available on kaggle.
